# Study of POSS on the Properties of Novel Inorganic Dental Composite Resin

**DOI:** 10.3390/polym12020478

**Published:** 2020-02-20

**Authors:** Jiahui Wang, Yizhi Liu, Jianxin Yu, Yi Sun, Weili Xie

**Affiliations:** 1 College of materials, Shanghai Dianji University, Shanghai 200000, China; 2 Department of Astronautic Science and Mechanics, Harbin Institute of Technology, Harbin 150000, China; 3 School of Materials Science and Engineering, Harbin Institute of Technology, Harbin 150000, China; 4 Department of Stomatology, Harbin Medical University, Harbin 150000, China

**Keywords:** dental composite resin, POSS, shrinkage, hardness, strength

## Abstract

Various amounts of methacryl polyhedral oligomeric silsesquioxane (POSS) were explored to be incorporated into novel nano SiO_2_ dental resin composites using light curing method. The scanning electron microscopy (SEM), optical microscopy, fourier transform infrared spectroscopy (FTIR), nanoindentation, nanoscratch and three-point flexure tests were performed. The volumetric shrinkage and mechanical properties such as hardness, elastic modulus, resistance, flexural strength and fracture energy were analyzed. With the additions of POSS, the volume shrinkage decreased and the mechanical properties initially increased. The effects of POSS on these properties were studied to provide a reference for clinically selecting a composite resin with excellent properties.

## 1. Introduction

Currently, the composite resin is the most important repairing material for tooth defects. Because of its many advantages such as color, strong plasticity, resistance to dissolution, easy polishing, simple operation, and close to the coefficient of thermal expansion, it is mainly used for fillings and restoration of dental caries and plays a pivotal role in dental restoration [1,2,3]. However, some of its shortcomings such as poor strength, bad abrasion resistance, and large polymerization shrinkage are also exposed as the service time increases. The application of new nanofillers in dental resins has been widely studied to improve the mechanical and anti-caries properties [4,5,6].

Polyhedral oligomeric silsesquioxane (POSS) is an organic–inorganic hybrid nanomaterial [7,8]. The composite resin with POSS as a filler has good biocompatibility and mechanical properties [9,10,11,12,13,14]. POSS is represented by the unique formula (RSiO_3/2_)_n_, where n is the number of the silicon atoms of the cage (1–3 nm in size) surrounded by organic corner groups (R). Due to its inorganic Si–O core cage structure and optional organic group chains, POSS can enhance matrix properties, such as mechanical strength [15,16,17], thermal stability [18,19,20] and oxidation resistance [21,22,23], and it can maintain good compatibility with the matrix in micro or nano scales. Meanwhile, POSS used in dental restoration can significantly improve the matrix on marginal adaptation [24,25] and ensure good biocompatibility and comfort level [4,26], which can greatly reduce the risk of micro effusion and secondary caries.

Ogliari et al. [27] simulated the shelf life to evaluate the stability of initiation systems on acidic photopolymerizable dental material through an experimental self-adhering flowable composite resin with bisphenol-A glycidyl methacrylate (Bis-GMA), tri-ethylene glycol dimethacrylate (TEGDMA), acidic monomer (GDMA-P), and inorganic fillers. Patnaik et al. [28] fabricated the resin matrix consisting of Bis-GMA, TEGDMA, camphorquinone (CQ) and ethyl 4 dimethyl amino benzoate (EDMAB), and evaluated mechanical and thermal properties and polymerization shrinkage with maximum conversion. Hao Fong et al. [29] chose methacryl POSS to incorporate into Bis-GMA for prepared novel dental restorative composites. The results showed that only a small percentage of methacryl POSS substitution of Bis-GMA in the resin systems could improve the mechanical properties of the composite. In our previous works [30,31], different contents of methacryl POSS were added into dental composite resins and BG (barium oxide glass powder) matrix dental composite resins, respectively, and some properties including polymerization shrinkage and mechanical behavior were evaluated.

In this work, various amounts of methacryl POSS were explored to be incorporated into novel nano SiO_2_ matrix dental resin composites using light curing method. The scanning electron microscopy (SEM), optical microscopy, fourier transform infrared spectroscopy (FTIR), nanoindentation, nanoscratch and three-point flexure tests were performed, and the effects of the contents of POSS on the volumetric shrinkage, hardness, elastic modulus, resistance flexural strength and fracture energy were studied.

## 2. Experimental 

**Figure 1:**
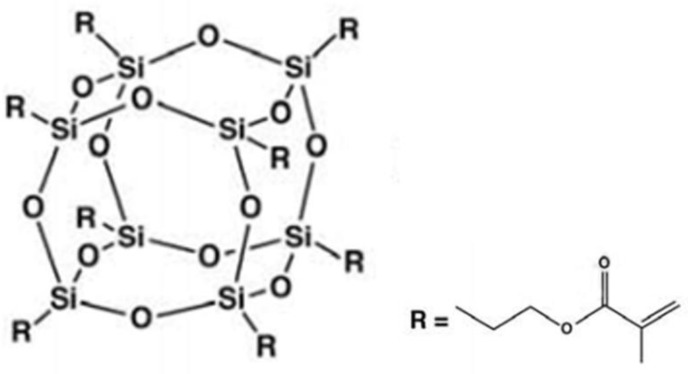
The molecule structure of methacryl polyhedral oligomeric silsesquioxane (POSS).

Bis-GMA (Bisphenol A glycerolate dimethacrylate) and TEGDMA (Tri(ethylenglycol) dimethacrylate, 98%) were purchased from Aldrich Chemical Co., Shanghai, China. The commonly used visible light photo-initiator CQ (camphorquinone, 97%) and co-initiator (2-(dimethylamino) ethylmethacrylate, DMAEMA, 98%) were selected for this research and purchased from Aldrich Chemical Co., Shanghai, China. The inorganic filler used in this study was surface modified KH-550 nano SiO_2_ with an average particle size of 30 nm, purchased from Deco Dokin Co. Shanghai, China. Surface modified KH-550 nano SiO_2_ is a white powder that mixes better with the matrix and provides better mechanical properties to the matrix compared with dental resin matrix without inorganic filler. All of the above materials can be used as a dental resin matrix. Methacryl POSS was obtained from Hybrid Plastics (Fountain Valley, Hattiesburg, CA, USA, Figure 1). The proportions of POSS hybrid inorganic dental composite resin and organic resin matrix were listed in Table 1 and Table 2.

### 2.1. Synthesis of Materials

An organic resin matrix solution containing Bis-GMA, TEGDMA, CQ and DMAEMA was prepared by mixing in a container kept away from light. Methacryl POSS was added to the solution of neat resins. Nano SiO_2_ was incorporated into the organic composite solution and magnetically blended uniformly in the vacuum allowing for the escape of air bubbles for 2 h. The mixture solution was poured into metal molds and the visible light curing method was applied. The curing time of each sample was 60 s at room temperature. The prepared samples were immersed in distilled water at 37 °C for 24 h and were stored in configured artificial saliva at 36.5 °C for 4 weeks. The sizes of the specimens were measured accurately and recorded immediately before testing.

### 2.2. Characterization

#### 2.2.1. FTIR Characterization 

Fourier transform infrared (FTIR) spectroscopy analysis was performed using the FTIR spectrometer (Avatar360, Nicolet, Madison, US). The samples involved in this part of the study were POSS and organic composite resins without inorganic filler.

#### 2.2.2. Morphology Characterization

A VHX-600E digital microscope (Keyence Company, Shanghai, China) was used for optical microscopy of the nanocomposite resins with a real-time depth composition, two/three dimensional functions, and 500–5000 zoom.

The fracture morphology of the POSS dental resin composite was characterized using a field-emission scanning electron microscope (FESEM, JEOL 7600F, Osaka, Japan).

#### 2.2.3. Shrinkage

The densities of uncured and cured resin specimens were measured by a pycnometer to determine polymerization shrinkage according to the Archimedes’ principle.

#### 2.2.4. Mechanical Properties Tests

The G200 Nano Indenter (Agilent Technologies, Santa Clara, CA, USA) was employed to study the hardness of the nanocomposite resins. The indenter used in the test was a diamond triangular pyramid Berkovich indenter (TB 20114 ISO). The maximum depth of 2000 nm was used on each sample. The loading speed was maintained at the same strain rate of 0.05 s^-1^. The data was averaged from six specimens during each test to get the most accurate results.

The G200 Nano Indenter was also used to measure the resistance of the nanocomposite by the nanoscratch testing. The indenter tip scratched on the sample with a wear load value of 200 mN. The scratch length and speed were set as 400 μm and 10 μm/s, respectively. Three independent scratch tests were carried out for each sample.

A T1-FR010TH A50 (ZWICK Materials Testing Machine, Ulm, Germany) was used to measure flexural strength by three-point flexure testing (according to ISO4049: 2000) at a crosshead speed of 0.5 mm/min. The specimens were prepared in stainless steel molds with a dimension of 2 mm by 2 mm × 25 mm in length.

A three-point flexure test was also used to measure the fracture energy, which is the dissipative outside energy during fracture. Fracture energy can be used as the measurement of the toughness of the materials.

## 3. Results and Discussion

### 3.1. FTIR Analysis

**Figure 2:**
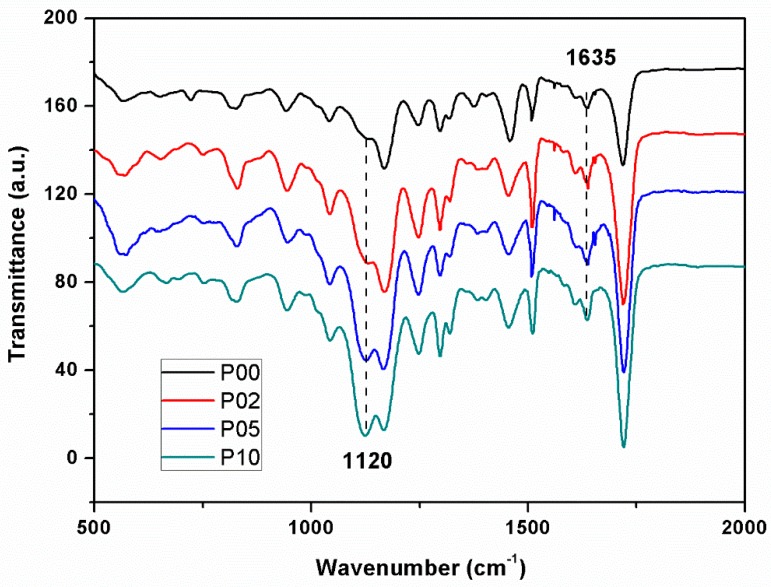
FTIR spectra of composite resins with different POSS additions.

The FTIR spectra of composite resins with different POSS additions are shown in Figure 2. The band at 1635 cm^-1^ was attributed to characteristic absorption peaks of C=C bond. After the curing reaction, the methacrylate double bonds had partly changed and the peak intensity decreased. The band at 1120 cm^-1^ is attributed to the stretching vibration of Si–O–Si in POSS. With the increasing addition of POSS, the peak intensity increased [32].

### 3.2. Low Volumetric Shrinkage

The volumetric shrinkage of POSS hybrid inorganic dental composite resin was evaluated. The volumetric shrinkage value of the dental resin matrix was 3.59%. With the increasing additions of POSS, the volume shrinkage decreased. The values were about 2.98%, 2.87% and 2.54% with the addition of 2%, 5% and 10% POSS, respectively. POSS reduced the shrinkage of resins obviously due to the limitation of free volume variation. Before curing, large chain monomers such as Bis-GMA and TEGDMA were dispersed in the matrix. After curing, the movement of chains was not limited by amount of cross-linked points and the free volume of resins changed remarkably. When the addition of POSS was incorporated in resins, due to its unique inorganic nanocage surrounded with organic groups, it can attract and even entwine monomer chains, which made monomers disperse more tightly before the reaction. The movement of chains is limited by plenty of cross-linked points. The unique nanocage structure of POSS limited the change of free volume of the resin and the volume of the nanocage of POSS did not change after the reaction.

### 3.3. Mechanical Properties

**Figure 3:**
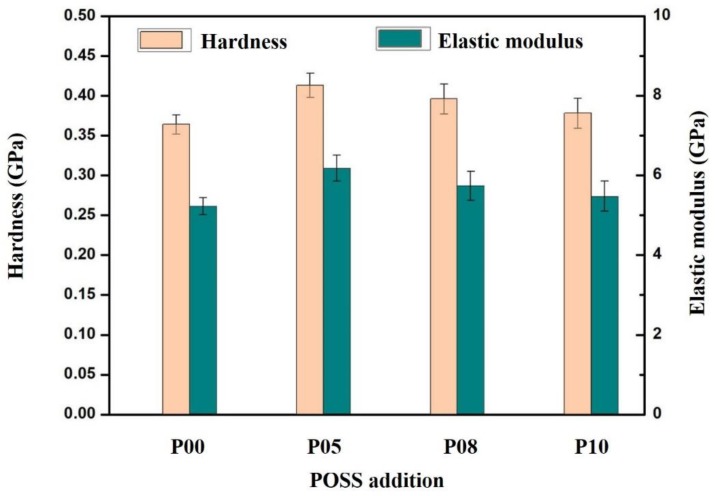
Hardness and elastic modulus of composite resins with different POSS additions.

**Figure 4:**
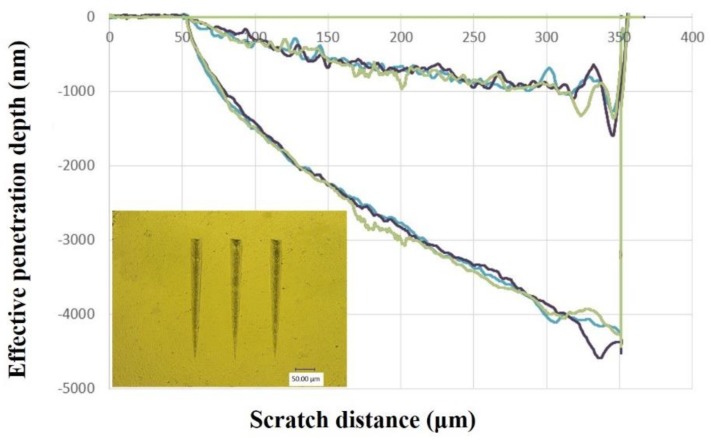
The effective penetration depth curves of neat dental resin matrix under load.

**Figure 5:**
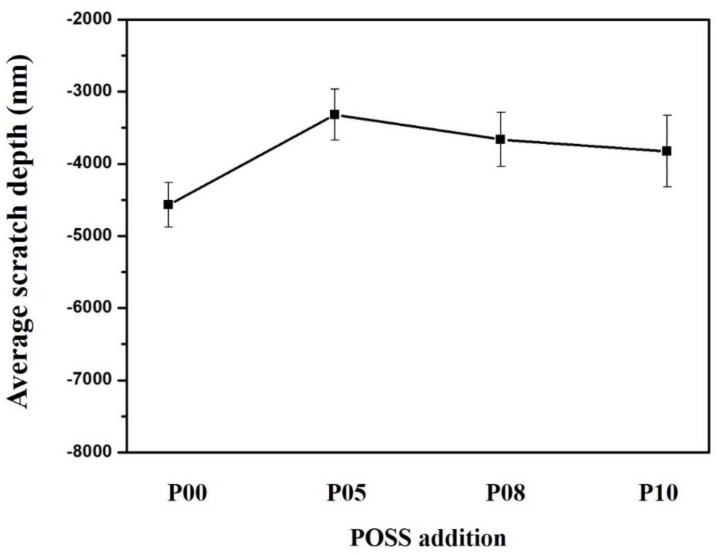
Average scratch depth of composite resins with different POSS additions.

The small addition of POSS remarkably enhanced the hardness and scratch resistance of the dental resin matrix. The hardness and elastic modulus of composite resins with different POSS additions were shown in Figure 3. The effective penetration depth curves of neat dental resin matrix under the load and average scratch depth of composite resins with different POSS additions were shown in Figure 4 and Figure 5, respectively. Compared with typically used fillers, a low amount of POSS can increase mechanical properties [12,33]. As the POSS addition increased, the nanoparticles and the resin chains attracted tightly and formed big polymer particles, which was benefit to stress transfer. The mechanical properties of the composite initially increased. When it exceeded 5 wt%, the heterogeneous distribution and stress concentration appeared. The mechanical properties of the POSS composite resin reduced. These properties showed a similar variation trend according to the percentage of POSS, which indicates that POSS have the same influence on the composite matrix. Meanwhile, compared with the pure organic matrix and dental matrix with barium oxide glass powder [28,29], the mechanical properties of matrix containing with nano SiO_2_ are also improved obviously.

**Figure 6:**
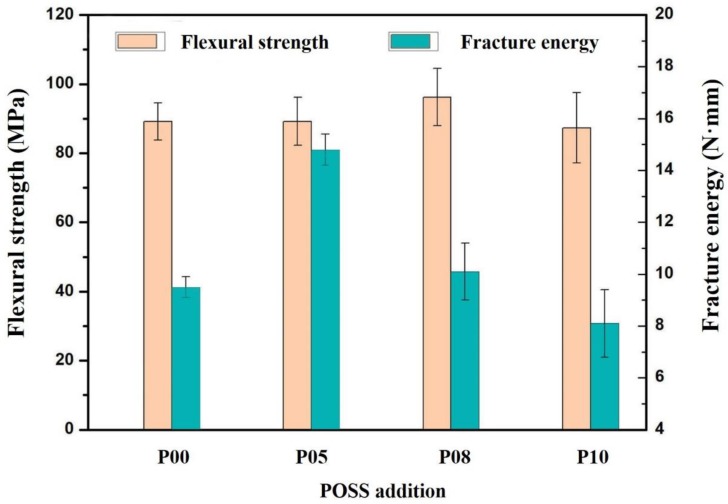
Flexural strength and fracture energy of composite resins with different POSS additions.

**Figure 7:**
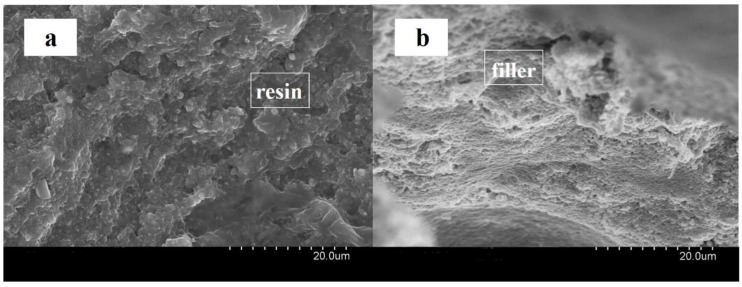
Scanning electron microscopy (SEM) images of the fracture topography of (**a**) organic dental composite resin and (**b**) POSS hybrid dental composite resin.

Flexural strength and fracture energy can reflect the ability to withstand complex loads for dental restorations. The results of flexural strength and fracture energy of composite resins with different POSS additions were shown in Figure 6 and SEM images of the fracture topography of the organic dental composite resin and POSS hybrid dental composite resin were shown in Figure 7. When the addition of 2 wt%, POSS increased the flexural strength and fracture energy. POSS monomers polymerized with the matrix to form a cross-linked net framework. POSS can be helpful to increase the toughness of resins. The formation of a cross-linked polymeric network with more POSS was obvious. The flexural strength and fracture energy of the POSS dental resin matrix decreased sharply. POSS has a unique microparticle surface structure, which is closely bonded to the resin matrix interface. The addition of a small amount can make the material have excellent mechanical properties compared with ordinary composite materials. Meanwhile, the interface layer formed between the modified nano SiO_2_ particles and the POSS organic–inorganic molecule has higher strength. Modification of nano SiO_2_ with a coupling agent can reduce particle agglomeration. As a result, the distribution of nano SiO_2_ is more uniform and the effect of reinforcing and toughening is also obvious.

The POSS particles can increase the properties of the composite resin matrix. The big size effect of the particles can cause stress concentration under loading. The resin matrix around the organic and inorganic materials produces yielding phenomena including cavitation, shear zones and crazing. POSS particles can prevent cracks to a certain extent or terminate crack blunting and crack propagation. A lot of energy is consumed, and the craze damage further develops into cracks under the effect of stress. The mechanism of transition between cracks and cracking damage may be due to the structural defects of the polymer. When the polymer is affected by various external forces, stress concentration and cracking damage occurred [34,35,36]. If no nanoparticles are embedded in the resin material, the craze will gradually develop into the destructive crack which will lead to fracture of the material. However, with the enforcement of nanoparticles, they are able to enter the interior of the gap to form a filamentous connection structure. The filamentous connection delays the fracture of the resin material, so that more external energy is consumed for the material to fracture. The toughness and strength of the material are thus improved.

However, once the content of nanoparticles exceeds a certain limit, the internal gaps between cracks will be smaller than the diameter of the clumps. This results in the nanoparticles becoming unable enter the gap and the crack cannot return to the state of craze. The material produces a larger gap or deformation under the impact forces and even generates macroscopic cracks. Therefore, the nanoparticles tend to cause the impact strength and toughness of the resin material to decrease when the stress concentration occurs. In addition, the existence of particles prevents the crack from fracturing. Therefore, the addition of POSS fillers should be within a certain limit or it will have an adverse effect. Since the nano SiO_2_ and POSS in the composite resin are both nano-scale. They can be distributed uniformly and also expand the interface region between the matrix and the filler [37]. The resin and nanofiller interact at the interface and exhibit physical cross-linking or a nano effect, which transfers the stress and increases the mechanical properties.

Meanwhile, the mechanical properties of resin materials are closely related to its curing degree. The effect of curing is related to light intensity, time and color of the resin. The addition of inorganic filler will affect its light transmittance, which further reduces the curing effect. Meanwhile, the POSS composite resin is viscous. When it is injected into the mold, it may form tiny bubbles, which will cause a tiny void in the resin and decrease the mechanical properties of the resin.

## 4. Conclusions

In this work, various amounts of methacryl POSS were explored to be incorporated into novel nano SiO_2_ dental resin composites using light curing method. The volumetric shrinkage and mechanical properties such as hardness, elastic modulus, resistance, flexural strength and fracture energy were analyzed and the effects of POSS on these properties were studied.

With the additions of POSS, the volume shrinkage decreased remarkably. The values were about 2.98%, 2.87% and 2.54% with the addition of 2%, 5% and 10% POSS, respectively. POSS reduced the shrinkage of resins obviously due to the limitation of free volume variation.

As for the mechanical properties, the POSS hybrid inorganic dental composite resin exhibited a positive reinforcement effect when a small amount of POSS was added. When it exceeded 5 wt%, heterogeneous distribution and stress concentration appeared. The mechanical properties of the POSS composite resin reduced. The big size effect of the particles can cause stress concentration under loading. With the enforcement of nanoparticles, they were able to enter the interior of the gap and interface region to exhibit physical cross-linking or a nano effect, which transfers the stress and increases the mechanical properties.

## Figures and Tables

**Table 1:** The proportion of POSS hybrid inorganic dental composite resin.

Composite Code	Dental Resin Matrix		POSS
	Organic Resin Matrix Wt%	Nano SiO_2_	
P00	40	60	0
P02	38	60	2
P05	35	60	5
P10	30	60	10

**Table 2:** The proportion of organic resin matrix. Bis-GMA: bisphenol-A glycidyl methacrylate; TEGDMA: tri-ethylene glycol dimethacrylate; CQ: camphorquinone; DMAEMA: 2-(dimethylamino) ethylmethacrylate.

Organic Resin Matrix, Wt%			
Bis-GMA	TEGDMA	CQ	DMAEMA
49.5	49.5	0.5	0.5
